# A novel treatment based on powder jet deposition technique for dentin hypersensitivity: a randomized controlled trial

**DOI:** 10.1186/s12903-023-03431-y

**Published:** 2023-09-27

**Authors:** Hiroki Hihara, Kuniyuki Izumita, Tetsuo Kawata, Ryo Akatsuka, Ryo Tagaino, Aki Kitaoka, Chie Kayaba, Koji Ikeda, Keiichi Sasaki

**Affiliations:** 1 Division of Advanced Prosthetic Dentistry, Tohoku University Graduate School of Dentistry, 4-1 Seiryo- machi, Aoba-ku, Sendai, Miyagi 980-8575 Japan; 2 Perioperative Oral Care Support, Tohoku University Hospital, 4-1 Seiryo-machi, Aoba-ku, Sendai, Miyagi 980-8575 Japan; 3 Otemachi Kawata Dental Clinic, 6-19 Otemachi, Aoba-ku, Sendai, Miyagi 980-0805 Japan; 4 Akatsuka Dental Clinic, 2838-1 Mawatari, Hitachinaka, Ibaraki 312-0012 Japan; 5 Division of Molecular and Regenerative Prosthodontics, Tohoku University Graduate School of Dentistry, 4-1 Seiryo-machi, Aobaku, Sendai, Miyagi 980-8575 Japan; 6 Department of Development Promotion, Clinical Research, Innovation and Education Center, Tohoku University Hospital, 1-1 Seiryo-machi, Aoba-ku, Sendai, Miyagi 980-8574 Japan; 7 Tohoku University Graduate School of Dentistry, 4-1 Seiryo-machi, Aoba-ku, Sendai, Miyagi 980-8575 Japan

**Keywords:** Hydroxyapatite, Medical device, Randomized control trial, Translational study, Biomaterial

## Abstract

**Background:**

This study aimed to evaluate the efficacy and safety of dentin hypersensitivity (DH) treatment using a newly developed device based on a powder jet deposition (PJD) technique that creates a hydroxyapatite (HAP) layer on the dentin surface, thereby alleviating the hypersensitivity. The effect of the PJD treatment was compared with that of conventional treatment using Teethmate Desensitizer (TMD; calcium-phosphate containing material with TTCP (Ca_4_(PO_4_)_2_O) and DCPA (CaHPO_4_)), which has been used clinically in Japan with well-confirmed effectiveness.

**Materials and methods:**

A randomized controlled trial was conducted including 35 patients who had symptoms of DH in two or more quadrants. Two test teeth were selected per patient (70 teeth in total) and randomly assigned to PJD or TMD treatment. The efficacy was evaluated using the improvement rate for air and scratch pain according to the scores obtained via visual analog scale 12 weeks after treatment. The safety assessment was performed focusing on gingival index (GI) and spontaneous pain. The t-test was used to analyze the non-inferiority of PJD treatment compared to TMD treatment.

**Results:**

The improvement rate of air pain was 69.0% for PJD and 69.7% for TMD. The improvement rate of scratch pain was 80.8% for PJD and 81.7% for TMD. Non-inferiority with a margin of 10% was not observed for both air and scratch pain. No change was observed in GI from baseline and the improvement rate of spontaneous pain for PJD was higher than that for TMD.

**Conclusion:**

Non-inferiority of PJD to TMD treatment was not observed in this study; however, it was not statistically demonstrated, and the results were thus interpreted as inconclusive. PJD did improve the DH symptoms, as did TMD. PJD’s therapeutic effect was most likely attributable to the deposition of a HAP layer on the tooth surface, which would alleviate hypersensitivity for at least 12 weeks without causing severe adverse events.

**Trial registration:**

UMIN-CTR. ID: UMIN000025022. date: 02/12/2016.

## Background

Dentin hypersensitivity (DH) is caused by the exposure of dentin because of defection of enamel or cementum [1]. It is defined as “an exaggerated sensitivity of vital dentine exposed to thermal, chemical and tactile stimuli” [2]. Transient sharp pain occurs when stimulation is applied to the area. Nearly 30% of Japanese are affected by hypersensitivity [3]. The most accepted theory regarding the pathogenesis of DH is hydrodynamic theory [4–6]. According to it, when stimulation is applied to the surface of the dentin, the flow of fluid occurs in the dentine tubules. This causes pressure and excites the free nerve endings in dental pulp [7–9]. Recently, odontoblasts might well play an important role in the pathogenesis mechanisms of DH [10–12]. These findings suggest that chemical mediators are released from odontoblasts despite the absence of physical synapses, and paracrine cell-cell communication plays an important role in the occurrence of DH [13]. In any case, external environmental stimuli caused DH, DH treatment is thus focused on obturating the dentine tubules [14]. There are various methods for this such as crystal/salt precipitation and sealing with laser irradiation or resin materials [15–17]. However, crystals of fluoride, potassium nitrate, and calcium hydroxide show low sealing properties for dentine tubules [18–20]. Additionally, the long-term effectiveness is uncertain, and retreatment is sometimes required [21].

**Fig. 1:**
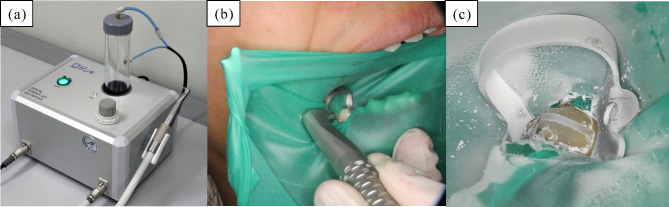
Photographs of PJD-UNIT (a) main body and handpiece of PJD-UNIT, (b) application of spraying on the tooth, (c) after application of PJD-UNIT (after injection test)

To solve these problems, we focused on the powder jet deposition (PJD) technique [22–25] as a new method for treating DH. PJD is a method whereby a thin layer of hydroxyapatite (HAP) is formed on the material surface by injecting fine HAP particles at high speed. The HAP layer can be formed on the tooth surface using the PJD technique. The formation of the HAP layer is due to particles with high kinetic energy colliding with the substrate, and some particles are plastically deformed and repeatedly attached to the substrate [26, 27]. We have developed a medical device named PJD-UNIT (SANGI CO., LTD. Japan) that can efficiently form a layer of HAP on the tooth surface **(**Fig. 1**)**. PJD UNIT consisted of dedicated HAP powder and the device to deposit it on dentin at a high speed. PJD-UNIT consisted of two parts: a deposition control device and a handpiece. The HAP layer adhered as firmly as composite resin bonded to the tooth surface. In addition, the micro-Vickers hardness and corrosion resistance were equivalent to those of enamel [26]. Furthermore, the HAP layer after the thermal cycle assuming oral environment showed superior microstructural and mechanical properties [28]. In addition, the HAP layer formed on the dentin reduced the permeability of dentin tubules more than with commercially available dentin desensitizers [29]. As the HAP layer formed by the PJD-UNIT has the same components as a tooth and the dentine tubules are filled with HAP, it has no harmful effects on the human body [29]. HAP layer is transparent and inconspicuous, it does not cause aesthetic problems. Therefore, the effect of treatment by PJD-UNIT is expected to be the same as or better than that of conventional treatment. Previous studies have confirmed that no major damage occurred [26, 28, 29].

In addition, we confirmed the safety of soft and hard tissues in Beagle dogs (approval number 19MedA-1 6: Committee of the Tohoku University Environmental & Safety Committee) based on consultation with Japanese regulatory authorities (e.g., the Pharmaceutical and Medical Device Agency). Based on these results, we obtained certification for the implementation of clinical trials. Therefore, we previously conducted an exploratory clinical trial to determine the effectiveness and safety of the HAP layer formed by the PJD-UNIT in treatment of dental caries, DH, and discolored teeth [30]. Based on the results, in the treatment of DH, the main symptoms of air pain and cold-water pain decreased according to the Numerical Rating Scale (NRS) score after 4 weeks compared with before treatment. In addition, no harmful effects were observed in dental pulp. Although non-serious adverse events were reported in 2 patients, they were not attributable to the PJD-UNIT or device malfunction.

Based on the results of preclinical studies and exploratory clinical study, the effectiveness and safety of the PJD-UNIT for DH were demonstrated. Nevertheless, as the exploratory clinical trial was a single-arm trial with a limited number of subjects, both the clinical effectiveness and safety were insufficient in terms of obtaining pharmaceutical approval. As mentioned above, the PJD-UNIT may have advantages in DH treatment over conventional methods because better sealing of dentinal tubules with high biocompatible HAP can be achieved [29, 30]. Thus, if the therapeutic effect of the PJD-UNIT is not worse than conventional treatments, it is clinically valuable. Therefore, we conducted a non-inferiority randomized controlled trial to evaluate the efficacy and safety of DH treatment using the PJD-UNIT compared with that of a commercially available agent, Teethmate Desensitizer (TMD; Kuraray Noritake, Japan) [31]. TMD contains calcium phosphate, tetracalcium phosphate [TTCP; Ca_4_(PO_4_)_2_O], and dicalcium phosphate anhydrous (DCPA; CaHPO_4_), the combination of which could spontaneously transform to hydroxyapatite [HAP; Ca_10_(PO_4_)_6_(OH)_2_] [32]. The HAP obturates the dentine tubules. Since this is a study to obtain pharmaceutical approval in Japan, it is necessary to compare with materials that have pharmaceutical approval. TMD has been approved in Japan, and the efficacy of the latter has been scientifically demonstrated [31–38], with long-term sustainability especially demonstrated in vitro [32]. Additionally clinical studies show their effectiveness and safety [31, 35–38]. Therefore, it was employed as a representative conventional treatment (i.e., an active control).

## Methods

### Study design

This study was a single-blind, multi-center (Tohoku University Hospital and Otemachi Kawata Dental Clinic) randomized controlled trial with a split-mouth design to compare the effect of PJD and TMD treatments. The protocol was reviewed and approved by the Institutional Review Board of Tohoku University Hospital (reference No. 163,006). In addition, this study and the clinical trial was performed in accordance with the Declaration of Helsinki, the Consolidates Standards of Reporting Trials (CONSORT), and the Good Clinical Practice (GCP) guideline. Before starting this study, we consulted the Pharmaceutical and Medical Device Agency, a regulatory authority in Japan, and obtained clinical trial notifications. The clinical trial was registered on the University hospital Medical Information Network (ID: UMIN000025022. date of first registration: 02/12/2016).

The patient inclusion criteria were as follows: (1) age ≥ 20 years; (2) informed consent signed by the patient; (3) subjects who were able to visit the hospital throughout the clinical trial period; (4) presence of DH symptoms in two or more quadrants with a visual analog scale (VAS) score of 30 mm or larger when air is applied at a pressure of 0.4 ± 0.05 MPa and a flow rate of 15 ± 3 L / min; and (5) subjects who agreed not to receive DH treatment until completion of the clinical trial even if they had DH symptoms in a tooth adjacent to the test tooth. The exclusion criteria were as follows: (1) presence of oral mucosal disease; (2) medical history of adverse reactions to local anesthesia; (3) pregnancy or possibility thereof; (4) participating in another clinical trial; (5) having other pain in an oral cavity; (6) taking pain medication; (7) subjects considered unsuitable for the trial by the investigators; and (8) presence of dental caries, pulp pain, or periodontitis with a probing pocket depth of ≥ 5 mm and bleeding on probing in a test quadrant.

### Random allocation

**Fig. 2:**
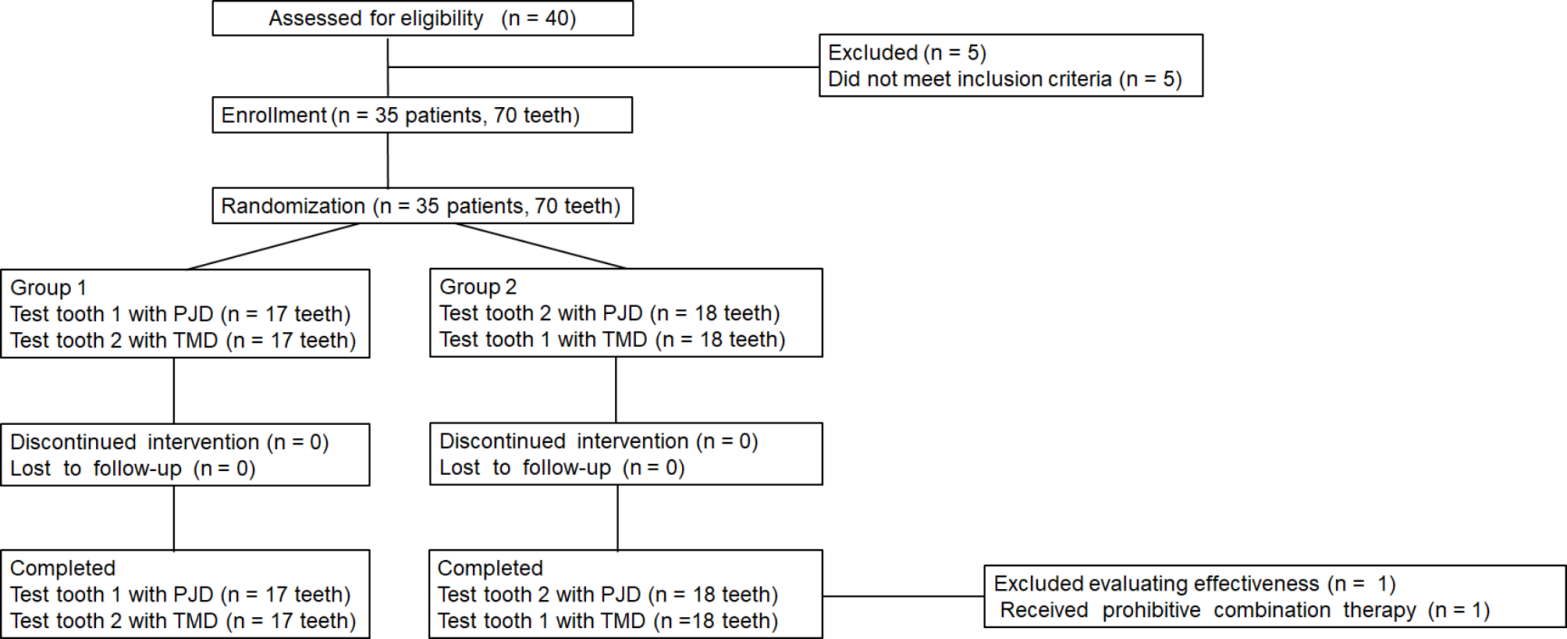
Flowchart of treatment

Patients who visited Tohoku University Hospital or a private dental clinic with symptoms of DH were asked to enroll in this clinical trial. In total, 40 patients consented to be assessed for eligibility. After written informed consent was obtained from the patient, the baseline VAS examination was performed by the examiner. As stated in inclusion criteria (4), test teeth were selected using split-mouth design. We examined the tooth that a subject felt was the most painful in daily life, and if the VAS value was 30 mm or greater, the tooth was designated as “Test tooth 1.” We chose “Test tooth 2” in order of priority: left–right opposite side, left–right upper–lower opposite side, and upper–lower opposite side of “Test tooth 1.” If “Test tooth 1” was the anterior part of the tooth, examination was performed on the anterior part, and if it was the molar part, examination was performed on the molar part. The tooth with the highest VAS value of 30 mm or greater was designated as “Test tooth 2.” The investigator then contacted the registration center and the test teeth were randomly allocated to one of the two treatments (i.e., PJD or TMD). The random allocation was performed using an interactive web response system. Figure 2 shows the CONSORT flow chart of the clinical trial. “Test tooth 1” with PJD was assigned to Group 1; “Test tooth 2” with PJD was assigned to Group 2. Assignment results were only available for the operator, and the patient and evaluator were blinded.

### PJD

The PJD-UNIT and HAP powder was provided by SANGI CO., LTD. in accordance with a joint research contract with Tohoku University. Test treatment was performed by operators. We selected three blinded evaluators who were not involved in the test treatment. Following the baseline examination and randomized allocation, the test teeth were cleaned using a rotary toothbrush with rubber cup by operator. Then, to blind the patient, the test tooth was subjected to both dummy and test treatments.

For PJD treatment, a dummy treatment was provided first. The dummy treatment was performed by application of water, instead of TMD, using an applicator brush for 40 s. The tooth was then isolated using a rubber dam for PJD treatment. The tooth was sprayed with HAP powder using the PJD-UNIT for 1 min under the following conditions: supply pressure, 0.5 MPa; powder fill volume, 5 g; and nozzle scan speed, approximately 5 mm/s; HAP particle. The distance between the tip of the spray nozzle and tooth surface was kept at about 3 mm within an allowable range of 1–5 mm. The excess powder was vacuumed using extra and intra oral suctions. Finally, the HAP layer formed on the tooth surface was polished using a diamond paste (Diamond Polisher Paste, GC, Japan).

### TMD

For TMD treatment, TMD was applied to the tooth surface using an applicator brush for 40 s. A dummy treatment was then performed. The tooth was isolated as described above and HAP powder was sprayed using the PJD-UNIT onto the rubber dam, instead of the tooth surface, under the same conditions as described above. Polishing using the diamond paste was performed on the non-treated area of the tooth after the dummy treatment.

### Evaluation

Evaluations were performed by only blinded evaluators. Operators were not involved in the evaluation. Pulp symptom (pain) caused by air application with inclusion criteria (3) was defined as “air pain” and by scratch using a dental explorer as “scratch pain.” The evaluators performed calibration before the evaluation. In case of air pain examination, air pressure and flow rate were controlled to maintain these levels using a PJD-UNIT syringe connected to an air pressure and flow rate controller. The airflow was checked chronologically using an airflow sensor. The unit syringe was held with the tip about 1 cm away from the target area, and air was applied with a pressure of 0.4 ± 0.05 MPa and a flow rate of 15 ± 3 L/min. In case of scratch pain, the evaluators were trained using a pressure gauge to ensure that the scratch force was constant. The tip of an explorer (Multi, YDM Corporation) was run with a pressure of about 20 g both ways for about 1 s.

“Spontaneous pain” for each subject tooth and the presence of pain that interferes with daily life were recorded. VAS for air, scratch, spontaneous pain and gingival index (GI) were recorded by the blinded evaluators the next day, and 1, 4, 8, and 12 weeks after treatment. During the follow-up period, adverse events were recorded, and their severity was determined based on the National Cancer Institute’s Common Terminology Criteria for Adverse Events (CTCAE), v4.0.

This study’s primary endpoint was improvement rate of air pain 12 weeks after treatment. The secondary endpoint was improvement of scratch pain 12 weeks after treatment. Safety endpoints were adverse events (spontaneous pain, GI, other adverse events) and device malfunction.

### Statistical analysis

Sample size calculation was performed as follows: VAS was used as an evaluation item in this clinical trial, whereas the NRS was used in the exploratory clinical trial. We therefore converted the NRS value to the VAS value (NRS value 10 → VAS value 100). The improvement rate of PJD treatment was estimated as 71.3 ± 28.2 [30]. Additionally, the improvement rate of TMD treatment was estimated as 60.7 ± 13.6 [31]. Based on these estimations, the mean air pain improvement rate of the PJD treatment group was set as 70.0%, the mean air pain improvement rate of the TMD treatment group was set as 60.0%, the common standard deviation was set as 30.0, and the non-inferiority margin was set as 10.0. The required number of cases was calculated to be 28, and 35 was set when considering dropout.

The improvement rate of air pain was calculated as follows:

$$\text{I}\text{m}\text{p}\text{r}\text{o}\text{v}\text{e}\text{m}\text{e}\text{n}\text{t} \text{o}\text{f} \text{a}\text{i}\text{r} \text{p}\text{a}\text{i}\text{n} \left(\text{\%}\right)=\frac{\text{V}\text{A}\text{S} \text{b}\text{e}\text{f}\text{o}\text{r}\text{e} \text{t}\text{r}\text{e}\text{a}\text{t}\text{m}\text{e}\text{n}\text{t} - \text{V}\text{A}\text{S} \text{a}\text{f}\text{t}\text{e}\text{r} \text{t}\text{r}\text{e}\text{a}\text{t}\text{m}\text{e}\text{n}\text{t} }{\text{V}\text{A}\text{S} \text{b}\text{e}\text{f}\text{o}\text{r}\text{e} \text{t}\text{r}\text{e}\text{a}\text{t}\text{m}\text{e}\text{n}\text{t}}\times 100$$

Assuming the average improvement of air pain of the PJD group as µ_pjd_ and that of the TMD group as µ_tmd_, the hypothetical structure of the non-inferiority test was as follows:

H0: µ_pjd_ = µ_tmd_-10

H1: µ_pjd_ > µ_tmd_-10

Non-inferiority was verified by unpaired t-test.

For scratch pain, non-inferiority was verified by unpaired t-test using a temporary structure similar to the primary endpoint.

For adverse events determined to be related to the PJD-UNIT, the incidence rate was calculated for each treatment. The incidence rate for PJD and TMD treatments were statistically compared using Fisher’s exact test.

## Results

This study was conducted from January 2017 to September 2017. Forty patients provided written informed consent, and 35 patients met the inclusion criteria. We excluded a subject who received prohibitive combination therapy after completing a clinical trial, evaluating its effectiveness. Therefore, evaluation of effectiveness was performed for 34 patients and safety evaluation was performed for 35 patients. The mean age of the patients was 32.9 ± 14.2 years.

**Fig. 3:**
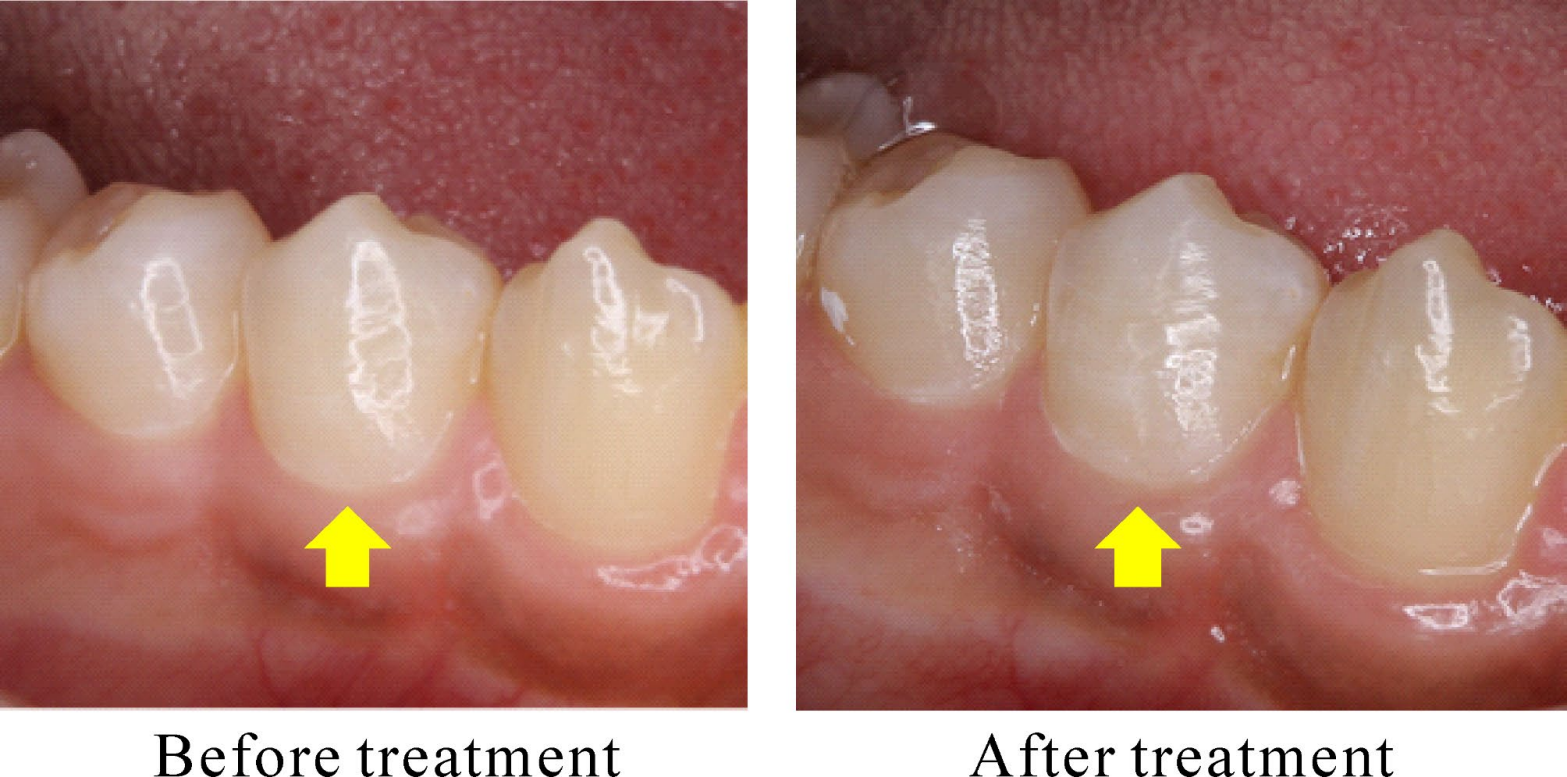
Before and after treatment with PJD-UNIT. Yellow arrows show the test tooth. PJD treatment was performed on cervix. HAP layer is transparent

Figure 3 shows the tooth before and after treatment with the PJD-UNIT. Table 1 shows the VAS and air pain improvement rate results. The air pain improvement rate was 69.0% for PJD treatment and 69.7% for TMD treatment. Although the mean improvement rates of air pain (primary outcome) were similar in both groups, PJD failed to meet the non-inferiority criteria (*p* = 0.196). Table 2 shows the VAS and scratch pain improvement rate results. The scratch pain improvement rate was 80.8% for PJD treatment and 81.7% of TMD treatment. As in the case of primary outcome, the null-hypothesis of non-inferiority test regarding the improvement rate of scratch pain was not rejected (*p* = 0.247).

**Table 1:** Change in VAS and improvement rate of air pain

	PJD		TMD		p
	n = 34		n = 34		
	VAS	improvement rate	VAS	improvement rate	
baseline	55.5 ± 13.6 (34)		55.6 ± 16.9 (34)		
Day 1	27.1 ± 23.2 (34)	52.4 ± 36.2 (34)	22.9 ± 22.5 (34)	58.8 ± 33.2 (34)	
1 week	26.5 ± 24.5 (34)	54.6 ± 35.0 (34)	20.4 ± 21.7 (34)	65.0 ± 29.8 (34)	
4 weeks	21.7 ± 22.2 (34)	62.1 ± 33.9 (34)	18.2 ± 18.4 (34)	68.4 ± 25.9 (34)	
8 weeks	20.3 ± 23.2 (34)	64.1 ± 39.6 (34)	19.3 ± 21.5 (34)	67.9 ± 29.4 (34)	
12 weeks	17.7 ± 20.3 (34)	69.0 ± 30.9 (34)	18.1 ± 20.8 (34)	69.7 ± 28.1 (34)	0.196

**Table 2:** Change in VAS and improvement rate of scratch pain

	PJD		TMD		p
	n = 34		n = 34		
	VAS	improvement rate	VAS	improvement rate	
baseline	26.9 ± 19.9 (34)		26.6 ± 19.9 (34)		
Day 1	9.1 ± 15.4 (34)	68.4 ± 37.3 (28)	9.8 ± 15.7 (34)	66.9 ± 40.6 (28)	
1 week	9.6 ± 15.3 (34)	60.3 ± 77.3 (28)	8.4 ± 13.4 (34)	71.4 ± 31.7 (28)	
4 weeks	6.5 ± 10.8 (34)	78.3 ± 22.6 (28)	6.1 ± 10.0 (34)	78.7 ± 26.0 (28)	
8 weeks	6.2 ± 10.3 (34)	82.6 ± 22.1 (28)	5.9 ± 8.5 (34)	73.7 ± 45.1 (28)	
12 weeks	5.3 ± 9.3 (34)	80.8 ± 26.8 (28)	3.9 ± 5.4 (34)	81.7 ± 31.4 (28)	0.247

No severe adverse events or device malfunctions were observed. Minor adverse events were reported in 4 patients, with 2 developing stomatitis and 2 developing gingivitis (NCI CTCAE Grade 1). All of the patients recovered without treatment. We did not perform statistical analyses, as the number of cases was too small. Regarding spontaneous pain, the improvement rate was 89.5% for PJD treatment and 71.2% for TMD treatment. In addition, no change from baseline was observed in GI.

## Discussion

This randomized controlled trial failed to demonstrate non-inferiority of PJD treatment to TMD treatment. However, according to the mean improvement rate of DH symptoms, PJD treatment was as effective as TMD treatment.

This study was conducted according to the guidelines for clinical trials of DH, and the endpoints were set following the guideline [39]. It was suggested that the first choice for evaluation of DH would be air pain, and the second would be scratch pain (or thermal pain) [39]. In addition, a previous clinical study testing TMD used both air and scratch pain to evaluate the efficacy of the agent [30]. Therefore, we also employed air and scratch pain as endpoints in this clinical trial.

The improvement rate of air pain was 69.0% for PJD treatment, which was comparable to that reported in our previous study (71.3%) [30]. The improvement rate of scratch pain was 80.8% for PJD treatment and 81.7% for TMD treatment. These findings indicate that the HAP layer formed by the PJD-UNIT may have good stability, and thus it is effective in DH treatment. However, PJD treatment failed to meet the non-inferiority criteria with a margin of 10%, and the results were thus inconclusive. As we assumed a relatively large variance in the improvement rate of air pain to calculate the sample size as mentioned above, the reason for the failure to demonstrate non-inferiority might be related to other factors. One may be the relatively high improvement rate brought by TMD treatment in this study (71.3%), which was higher than that reported previously (60.7%) [31]. In addition, the baseline VAS of air pain was lower than the reported value [40, 41]. Therefore, one of the factors that made the effect of TMD treatment different from that reported was that the baseline VAS was slightly lower than the reported value.

It should be noted that VAS is complicated to use, especially with elderly people, and is associated with higher error rates [40]. In fact, there were some patients with high error rates in using VAS in this clinical trial. Although the evaluation methods may have been a factor, VAS is most commonly used for pain evaluation [42]. If a clearer explanation had been provided to the patients, the error rates in VAS might have been lower. Furthermore, the cause of DH was unclear due to the inclusion criteria of this study. DH was caused by enamel attrition and erosion, corrosion, abrasion, abfraction and soft tissue dehiscence [13]. Therefore, to reduce the influence of other factors, for the comparison, we grouped DH based on cause. In addition, to ensure that the groups had the same level of pain, it was necessary to stratify the teeth according to their initial VAS values. These are the limitations of this study. Therefore, it is also necessary to consider setting an upper limit for the VAS value. Moreover, new research must be conducted with comparatively stricter VAS inclusion criteria, an increased number of cases, and an extended evaluation period.

The reason why we failed to demonstrate non-inferiority of PJD compared with TMD was that the treatment procedure was performed more strictly than reported methods. We used a rubber dam on test teeth, whereas other studies performed the procedure with simple moisture sealing [31]. As mentioned in introduction, TMD is based on the principle that calcium phosphate is transformed into HAP [32]. HAP crystals grow when calcium phosphate reacts with calcium and phosphate in saliva [31, 32]. In addition, TMD may react with saliva and grow HAP crystals [32, 33] and the formation of HAPs could proceed in the presence of substrate, because saliva in the oral cavity is supersaturated with calcium and phosphate [32, 33]. Therefore, it is considered that the strict procedure of this trial increased the amount of TMD remaining on the dentin surface and promoted the formation of HAP crystals.

The TMD group showed a plateau state after 4 weeks, whereas the PJD group showed a sustained improvement trend throughout the entire observation period. The HAP layer formed by the PJD-UNIT was firmly deposited on dentin and some small HAP particles entered the dentine tubules [29]. This caused direct sealing of the dentinal tubules due to the high-speed collision and deeper penetration of the submicron particles generated by the collision into the dentinal tubules. It is suggested that HAP particles inside dentine tubules be mineralized [29]. In addition, exposing HAP to saliva also enhanced calcification [43, 44]. TMD also formed HAP immediately [32]. It was thus considered that TMD formed a HAP layer more rapidly than PJD. However, TMD only obdurate the dentine tubules [33]. On the other hands, the HAP layer formed by PJD has a thickness of 30–40 μm and is uniformly formed not only on the dentinal tubules but also on the entire surface [26]. In addition, the hardness of the HAP layer by PJD is as same as that of the enamel, and the bonding strength is the same as that of the composite resin and enamel [26, 29].

Akatsuka suggested that the dentinal tubules are a calcium-rich environment, and there is a strong possibility that HA particles deposited inside the tubules could enter a recalcification process that generates a strong and permanent adhesion between the biomaterial and the tooth [29]. Therefore, we estimated that the creation of the HAP layer by PJD treatment promoted greater and longer calcification of the HAP layer than TMD. PJD is thus considered to have a lasting effect of 12 weeks or more compared with TMD, and the long-term prognosis is equal to that of TMD. However, it is necessary to investigate this over a longer observational period. Therefore, this is another limitation of this study.

Although minor adverse events were observed, they were mild and did not require treatment. There are a few studies on adverse events encountered during DH treatment. It was reported that desensitizer containing a high concentration of calcium sodium phosphosilicate could cause gingival inflammation [42, 45]. In DH treatment using a laser, pulp symptoms were assessed. We therefore also assessed both GI and spontaneous pain as a representative pulp symptom. No deterioration was observed after PJD treatment regarding both GI and spontaneous pain. In fact, the improvement rate of spontaneous pain for PJD treatment was slightly better for PJD treatment than TMD treatment. Therefore, it can be concluded that the safety of DH treatment with the PJD-UNIT is clinically acceptable [46]. For PJD treatment, no deterioration was observed regarding the safety endpoint of spontaneous pain and GI. Additionally, according to a new theory, it has been reported that paracrine cell-cell communication is one cause of DH [13]. Therefore, we estimate that less irritating substances are useful. Since HA is composed of the same component as teeth, obturation of the HAP layer is safer and more useful than TMD. Furthermore, the improvement rate of spontaneous pain for PJD treatment showed an improvement tendency compared with TMD treatment. It was thus considered that the PJD-UNIT had sufficient safety for DH treatment.

## Conclusion

Non-inferiority of PJD to TMD treatment was not observed in this study; however, it was not statistically demonstrated, and the results were thus interpreted as inconclusive. PJD did improve the DH symptoms, as did TMD. PJD’s therapeutic effect was most likely attributable to the deposition of a HAP layer on the tooth surface, which would alleviate hypersensitivity for at least 12 weeks without causing severe adverse events.

## Data Availability

Restrictions apply to the availability of these data. Data were obtained from SANGI CO., LTD. and AMED and are available from the corresponding author on reasonable request and with the permission of SANGI CO., LTD. and AMED.

## Abbreviations

CONSORT: Consolidates Standards of Reporting Trials
DCPA: dicalcium phosphate anhydrous
DH: dentin hypersensitivity
GCP: good laboratory practice
GI: gingival index
HAP: hydroxyapatite
NRS: numerical rating scale
PJD: powder jet deposition
TMD: Teethmate Densensitizer®
TTCP: tetracalcium phosphate
UMIN: University hospital Medical Information Network
VAS: visual analog scale
