# Correction: A novel treatment based on powder jet deposition technique for dentin hypersensitivity: a randomized controlled trial

**DOI:** 10.1186/s12903-023-03664-x

**Published:** 2023-11-28

**Authors:** Hiroki Hihara, Kuniyuki Izumita, Tetsuo Kawata, Ryo Akatsuka, Ryo Tagaino, Aki Kitaoka, Chie Kayaba, Koji Ikeda, Keiichi Sasaki

**Affiliations:** 1 Division of Advanced Prosthetic Dentistry, Tohoku University Graduate, School of Dentistry, 4–1 Seiryo- machi, Aoba-ku, Sendai, Miyagi 980–8575 Japan; 2 Perioperative Oral Care Support, Tohoku University Hospital, 4-1 Seiryo-machi, Aoba-ku, Sendai, Miyagi 980-8575 Japan; 3 Otemachi Kawata Dental Clinic, 6-19 Otemachi, Aoba-ku, Sendai, Miyagi 980-0805 Japan; 4 Akatsuka Dental Clinic, 2838-1 Mawatari, Hitachinaka, Ibaraki 312-0012 Japan; 5 Division of Molecular and Regenerative Prosthodontics, Tohoku University Graduate School of Dentistry, 4-1 Seiryo-machi, Aobaku, Sendai, Miyagi 980-8575 Japan; 6 Department of Development Promotion, Clinical Research, Innovation and Education Center, Tohoku University Hospital, 1-1 Seiryo-machi, Aoba-ku, Sendai, Miyagi 980-8574 Japan; 7 Tohoku University Graduate School of Dentistry, 4-1 Seiryo-machi, Aoba-ku, Sendai, Miyagi 980-8575 Japan

**Correction: BMC Oral Health 23, 695 (2023)**

**https://doi.org/10.1186/s12903-023-03431-y**

In the original publication of the article [1], the author would like to add the missing grant number 22K17070. The revised Funding is given below.

This work was supported by Japan Agency for Medical Research and Development (AMED) Grant No. 16lm0103007j0005 and a Japan Society for the Promotion of Science (JSPS) Grant-in-Aid for Scientific Research < KAKENHI > (No. JP18K17094, 20K10027 and 22K17070).

## References

[1] Hihara H (2023). A novel treatment based on powder jet deposition technique for dentin hypersensitivity: a randomized controlled trial. BMC Oral Health.

